# Application of molecular genetics techniques for detecting deleted segmental aneuploidy in Williams Syndrome

**DOI:** 10.1186/1471-2164-15-S2-P34

**Published:** 2014-04-02

**Authors:** Afaf Magbooli, Etimad Huwait, Ibtessam R Hussein, Mohammed AlQahtani

**Affiliations:** 1 Diagnostic Genomic Medicine Unit (DGMU), King Abdulaziz University, Jeddah, KSA; 2 Faculty of Science, King Abdulaziz University, Jeddah, KSA; 3 Centre of Excellence in Genomic Medicine Research (CEGMR), King Abdulaziz University, Jeddah, KSA; 4 Faculty of Medical Sciences, King Abdulaziz University, Jeddah, KSA

## Background

William’s Syndrome (WS) is one of the most powerful models of human cognition and rare genetic disorder, the incidence of WS range between 1/7500 and 50,000 and is considered to be a segmental aneuploidy due to heterozygous deletion of a contiguous gene at the long arm of chromosome 7 at q11.23 [1]. The deletion size usually ranges between 1.5-1.8 Mb. At least 26 genes have been detected in the deletion region in WS patients [2,3]. The aim of this study was to determine the efficient method for detection of the microdeletion at chromosome 7q11.23 in patients with WS. This comparative study will help to determine the effective methods to detect the microdeletion at chromosome 7q11.23 in William’s syndrome.

## Materials and methods

The study included 29 patients referred to the DGMU with the provisional diagnosis of WS. Peripheral blood samples were taken after obtaining informative consent from the parents or patients’ guardian. We applied conventional Cytogenetic (G-banding technique), Molecular Cytogenetic (Fluorescent In-Situ Hybridization) and Real time PCR Techniques for detection of segmental Aneuploidy in Williams-Beuren Syndrome.

## Results

No deletions were detected by Karyotyping, however one patient had translocation chromosomes (18; 19)(q11.1;p13.3). FISH technique could detect microdeletion in chromosome 7q11.23 in 6/23 patients. However, qPCR could detect deletion in 9/23 samples. Furthermore, the size of deletion could be detected accurately using the qPCR technique.

## Conclusions

Real time PCR could be used to confirm the FISH results and to detect small deletions or duplications that could not be detected by FISH.
